# Tissue distribution and quantification of bovine leukemia virus proviral DNA in cows after a long-term infection with wild-type strains and the attenuated BLV vaccine strain

**DOI:** 10.3389/fimmu.2026.1850743

**Published:** 2026-07-15

**Authors:** Guillermo Suárez Archilla, Gerónimo Gutiérrez, Luc Willems, Karina Trono, Vanesa Ruiz

**Affiliations:** 1 Instituto de Investigación de la Cadena Láctea (INTA-CONICET), Estación Experimental Agropecuaria Rafaela, Rafaela, Santa Fe, Argentina; 2 Instituto de Virología e Innovaciones Tecnológicas (IVIT), Centro de Investigaciones en Ciencias Veterinarias y Agronómicas (INTA-CONICET), Buenos Aires, Argentina; 3 Gembloux Agro-Bio Tech, University of Liège, Liège, Belgium

**Keywords:** attenuated vaccine strain, bovine leukemia virus, chronic infection, cows, proviral load, tissue distribution, vaccine safety

## Abstract

Bovine leukemia virus (BLV) is a B-lymphotropic oncogenic retrovirus that establishes a persistent infection in cattle, with lymphosarcomas occurring in up to 10% of infected animals. This study aimed to investigate the *in vivo* distribution of BLV provirus in organs and muscle tissues of infected animals after a long-term infection and to evaluate the safety of an attenuated BLV vaccine strain. The study focused on three cows that were part of a larger trial initiated in 2010 designed to evaluate the performance of the attenuated BLV strain. After seven years of infection, one animal from each experimental group was euthanized: one naturally infected with the BLV strain circulating in Argentina (Arg), and two experimentally inoculated with either a Belgian wild-type cloned provirus (WT) or the attenuated pBLV6073DX vaccine strain (Vac). Samples from blood, organs and muscle tissues were analyzed by nested-PCR and quantitative PCR (qPCR). At euthanasia, proviral load (PVL) in blood was high in Arg and WT (452,767 and 75,151 copies/µg DNA, respectively), but extremely low in Vac (55 copies/µg DNA). BLV provirus was detected in all tissues of Arg and WT, including muscle, whereas in Vac it was only detectable at very low levels in the spleen and the precrural lymph node. These findings provide novel evidence of viral biodistribution during chronic infection and, although based on a limited number of animals, represent an essential proof of concept by providing key long-term safety data on the attenuated vaccine strain that contributed to its subsequent regulatory evaluation and deregulation.

## 1 Introduction

Bovine leukemia virus (BLV), the causative agent of enzootic bovine leucosis (EBL), is a deltaretrovirus that naturally infects cattle, water buffalo, sheep, zebu, yak and bison (1–8). Although BLV infection has been successfully eradicated in several countries, its prevalence remains high worldwide (9). In Argentina´s main dairy-producing regions, more than 90% of farms are infected, with individual prevalence ranging from 66% to 90% (10–13). A recent study in Argentine beef cattle reported BLV infection in 71% of the farms analyzed, suggesting gradual dissemination of the virus (14).

Most BLV-infected animals are asymptomatic carriers; however, in approximately one-third of cases, infection progresses to a non-malignant proliferation of B-lymphocytes, termed persistent lymphocytosis (PL). Only 0.1-10% of infected animals develop leukemia or lymphoma (15). The mechanisms underlying BLV-induced tumorigenesis remain poorly understood. Following infection, BLV integrates a DNA intermediate as a provirus, randomly and permanently, into the host cell genome. The virus primarily targets B lymphocytes, although cells of the monocyte/macrophage lineage can also be infected (16–18). Once integrated, the virus propagates mainly through polyclonal expansion of infected cells. Somatic mutations associated with genetic instability may then drive the expansion of transformed clones, ultimately leading to leukemia and/or lymphoma (15).

BLV infection causes substantial economic losses worldwide through both direct and indirect effects, including reduced milk yield, reproductive inefficiency, lymphoma-associated mortality, carcass condemnation and trade restrictions (19–23). In recent decades, progress has been made towards developing a safe and effective BLV vaccine. We previously described the effectiveness and safety of a live-attenuated vaccine strain against BLV (24). This recombinant BLV provirus carries deletions in sequences essential for pathogenesis, combined with mutations in genes involved in replication. This attenuated vaccine (pBLV6073DX) proved infectious, replicated at very low levels, and elicited a robust immune response. Its performance was further assessed in a high-prevalence dairy herd, where 28 of 29 vaccinated heifers developed sterilizing immunity over a four-year period. Importantly, the attenuated strain was not transmitted to sentinel animals or to offspring (24, 25).

The aim of the present study was to investigate the distribution of BLV provirus in different organs and muscle tissues following long-term infection with either wild-type strains or the attenuated vaccine strain pBLV6073DX, thereby contributing further evidence regarding the safety of the vaccine strain. By assessing tissues destined for human consumption, this work aims to provide preliminary insights into the food safety profile of the attenuated strain, thereby contributing further evidence regarding its long-term safety and suitability for field implementation.

## 2 Methods

### 2.1 Animals

Three Holstein cows from the experimental field of the Centro de Investigaciones en Ciencias Veterinarias y Agronómicas (CICVyA), Instituto Nacional de Tecnología Agropecuaria (INTA) were analyzed. These animals had originally been enrolled as heifers in a larger trial initiated in 2010 to evaluate the performance of the attenuated pBLV6073DX strain, which included more animals per group (26). During the study all the animals were housed together in a loose-housing yard with free stalls at an experimental facility belonging to the CICVyA-INTA. Animals were periodically examined by the veterinarian staff. Their BLV status was assessed by detecting BLV-specific antibodies by ELISA and BLV DNA by nested-PCR (nPCR) and qPCR. After seven years of infection, three animals (one per group) were euthanized as the final step of the approved experimental protocol. Given the exploratory nature of this specific analysis, this limited number of animals was utilized to perform a preliminary proof of concept, aimed at evaluating BLV tissue distribution from a food safety perspective.

The three animals were designated as follows: Arg, a naturally infected BLV-seropositive heifer; WT, experimentally infected with the Belgian wild-type cloned provirus pBLV344; and Vac, experimentally infected with the attenuated pBLV6073DX strain. The pBLV344 strain is a Belgian wild-type provirus cloned from BLV-induced tumors (27), whereas pBLV6073DX is isogenic to pBLV344 but carries a point mutation in the transmembrane protein gene (nucleotide 6073) and a partial deletion of the R3-G4 sequences (positions 6614 and 6997) (28, 29). WT and Vac heifers were inoculated subcutaneously with a single dose of each strain prepared by transient transfection of mammalian cells, as previously described (24). Briefly, a monolayer of cells in a 150 mm dish was transfected with 30 µg of each plasmid using FuGENE HD (Promega). Forty-eight hours post-transfection, lysate aliquots were preserved at -80 °C in the presence of 15% trehalose.

### 2.2 Ethical approval

The trial was conducted in compliance with ethical and welfare standards, as well as specific requirements for a genetically modified viral strain, under the authorization of the National Service for Animal Health and Food Quality of Argentina (SENASA, Expediente 227352/10). All procedures were performed according to protocols approved by the Institutional Committee for Care and Use of Experimental Animals (CICUAE; approval reference CICUAE INTA- CICVyA 35/2010). Institutional guidelines were strictly followed throughout the study.

### 2.3 Samples

Seven years after experimental infections, the animals were euthanized. They were stunned using a non-penetrating captive bolt and suspended in a vertical position (head-down) to allow complete exsanguination via incision of the carotid arteries and jugular veins. Necropsies were performed by primary care veterinarians and veterinary pathologists. Each animal was euthanized and necropsied on separate days, and organ and tissue samples were aseptically collected with particular attention to minimizing cross-contamination. From each organ and tissue, a small central portion was excised using a sterile disposable scalpel blade to avoid contact with superficial blood, placed in an Eppendorf tube and stored at -80 °C until use. Plasma and buffy coat fractions were obtained from whole blood by centrifugation at 1,500 g for 15 min and stored at -20 °C until analysis.

### 2.4 Detection and quantification of BLV antibodies

Plasma antibodies specific to the whole BLV particle were quantified using an in-house ELISA, as previously described (30). Briefly, ELISA plates were coated with complete BLV-particles as antigen that had been partially purified from fetal lamb kidney cells persistently infected with BLV (FLK-BLV) by centrifugation on a discontinuous sucrose cushion. Plasma samples were diluted 1:40 and added to the plate in duplicates. Antibody titers were determined by an end-point dilution assay using two-fold serial dilutions of plasma. After incubation and washing, a peroxidase-conjugated anti-bovine IgG was added to each well. The reaction was developed with 3´,3´,5´,5´-tetramethylbenzidine (TMB) and H_2_O_2,_ stopped with 1N H_2_SO_4,_ and absorbance was measured at 450 nm.

Normalized values were expressed as a sample-to-positive (S/P) ratio. A weak positive control serum (WPC) was used to calculate the ratio: the difference between the raw optical density of the WPC and a negative control was set to 100%, and all sample values were expressed as a percentage of this value. Positivity was defined using a 25% cut-off, according to previous validation studies (30, 31). Titers were reported as the reciprocal of the highest dilution above the cut-off.

### 2.5 Detection of BLV provirus and quantification of proviral load

Total genomic DNA was extracted from buffy coat and tissue samples using the High Pure PCR Template Preparation kit (Roche, Germany), following the manufacturer’s instructions. DNA concentrations were measured with a NanoDrop spectrophotometer (Thermo Fisher Scientific, USA), and DNA quality and purity were assessed prior to amplification to ensure suitability for downstream analyses. Samples were stored at -20 °C until use.

BLV proviral DNA was detected by nPCR using 100 ng of DNA as template and primers flanking the deleted region of the vaccine strain (outer forward: 5’-CTCACTTCTGCTTCACCATCC-3’; outer reverse: 5’-GGCAGGCATGTAGAGAGTGG-3’; inner forward:5’-TGGAAAGAACTAACGCTGACGG-3’; inner reverse: 5’- CCCCAACCAACAACACTTGCTT-3’), as previously described (32). Wild-type and pBLV6073DX proviruses were identified by visualization of 610-bp and 230-bp fragments, respectively. Each run included positive and negative internal controls, as well as no-template controls for both PCR rounds.

Proviral load (PVL) was quantified by SYBR Green real-time qPCR targeting the *pol* gene (32, 33). Each reaction contained Fast Start Universal SYBR Green Master Mix (Roche), 800 nM forward and reverse BLV *pol* primers (BLVpol5f: 5’-CCTCAATTCCCTTTAAACTA-3’; BLVpol3r: 5’-GTACCGGGAAGACTGGATTA-3’) and 100 ng of purified DNA. Cycling conditions were: 50 °C for 2 min, 95 °C for 10 min, followed by 40 cycles of 95 °C for 15 sec, 55 °C for 15 sec, and 60 °C for 1 min. All samples were tested in duplicate on a StepOne Plus instrument (Applied Biosystems). Positive, negative, and a no-template controls were included in each plate to verify assay performance. Amplicon specificity was confirmed by dissociation curve analysis.

For quantification, plasmid pBLV1 containing a BLV *pol* fragment (kindly provided by Jacek Kuzmak, NVRI, Pulawy, Poland) was used as a standard. Tenfold serial dilutions ranging from 1 × 10^6^ to 1 copy/µl were prepared to generate the standard curve and estimate BLV copy number per µg of DNA. Based on this dynamic range and DNA input, the analytical sensitivity of the assay was estimated at 50 copies per µg of total DNA. To minimize inter-plate variability, all PVL determinations (buffy coat and tissues) for each animal were performed on the same plate. To ensure reaction uniformity and control for tissue-specific variations in DNA extraction efficiency, a constant input of 100 ng of total DNA was used for all qPCR assays, and proviral loads were consequently expressed as copies per microgram (µg) of DNA. Since each sample was assayed in duplicate, the mean of the two values was considered as the copy number of the sample. Proviral load was considered high when exceeding 2,000 copies per µg of DNA, corresponding to approximately 1% of cells carrying BLV provirus (34).

## 3 Results

To investigate the distribution of BLV proviral DNA in chronically infected cattle, we analyzed different organs and tissues from three BLV-infected animals enrolled in a study evaluating the attenuated BLV vaccine. No clinical signs related to enzootic bovine leukosis or other health complications were recorded, and all cows remained asymptomatic during the 7-year observation period. As agreed with SENASA, the animals were euthanized at the conclusion of the experiment. The Arg cow was naturally infected with BLV, whereas the other two animals were experimentally inoculated with either the pBLV344 wild-type strain (WT) or the attenuated pBLV6073DX vaccine strain (Vac), respectively. After seven years of experimental infections, necropsies were performed to collect organ and muscle samples. No macroscopic lesions were observed in any of the infected animals.

**Figure 1:**
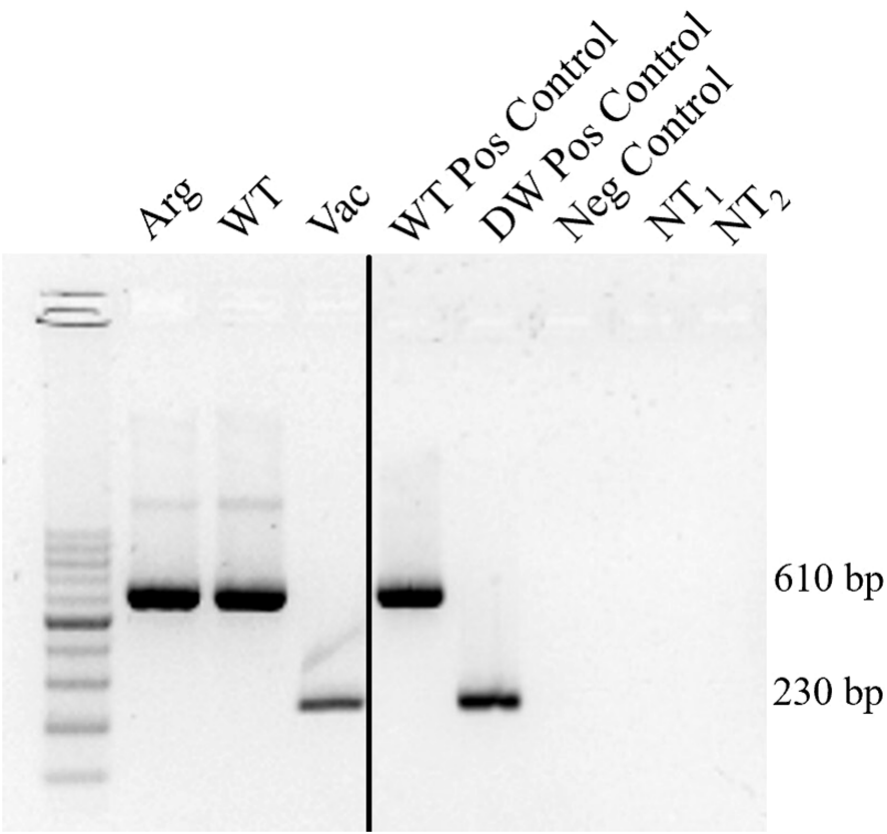
BLV proviral DNA detection by nPCR in the buffy coat of Arg, WT and Vac. The presence of the wild-type (WT) and the BLV6073DX (DX) strains were differentiated by the size of the amplicon. Positive and negative controls, as well as a no-template control for the first (NT1) and second round (NT2) of the nPCR were included. Samples were run on the same gel; the vertical black line indicates where non-adjacent lanes were spliced together for presentation purposes. Full-length unedited gel image is available in the **Supplementary Material**.

At euthanasia, BLV proviral DNA was detectable in peripheral blood (buffy coat) of all three animals by nPCR, distinguished by amplicon size (**Figure 1**). qPCR analysis revealed very high proviral loads in Arg and WT (452,767 and 75,151 copies/µg DNA, respectively), but extremely low levels in Vac (55 copies/µg DNA) (**Table 1**). Consistently, BLV-specific antibody titers were higher in Arg and WT (128 and 64, respectively) compared with Vac (32) (**Table 1**).

**Table 1:** Detection and quantification of BLV provirus and antibody titers in peripheral blood of infected animals.

Animal	Status	BLV provirus (Buffy coat)		Antibody titer (Plasma)
		nPCR	qPCR^a^	Elisa
Arg	Naturally infected (wild-type)	+	452,767 ± 18,481	128
WT	Experimentally infected (wild-type pBLV344)	+	75,151 ± 4,711	64
Vac	Experimentally infected (attenuated pBLV6073DX vaccine strain)	+	55*	32

(+): positive.

^a^ Values represent the mean ± standard deviation of proviral load (PVL) from qPCR duplicates, expressed as copies/µg DNA.

*The duplicate was below the linear dynamic range of the assay (<50 copies/µg DNA).

Analysis of organs and tissues showed BLV proviral DNA by qPCR in all samples from Arg and WT, with the highest loads in lymph nodes, spleen, liver and uterus (**Table 2**). In Arg, the spleen exhibited the highest PVL (250,552 copies/µg DNA), followed by lymph nodes (67,651-121,216 copies/µg DNA). All other organs had high PVL (>2,000 copies/µg DNA), except spinal cord and cerebellum. In WT, the spleen and lymph nodes also showed the highest PVL (45,191-53,014 copies/µg DNA), with additional high levels in liver, uterine body, bone marrow and heart. Proviral DNA was further detected in muscle tissues commonly consumed as meat with values ranging from 1,890 to 6,397 copies/µg DNA in Arg, and 710 to 9,353 copies/µg DNA in WT (**Table 2**). In contrast, no BLV proviral DNA was detected by nPCR in any organs or muscle tissues obtained from Vac. However, qPCR analysis revealed BLV proviral DNA in the spleen and precrural lymph node, at levels below the linear dynamic range of the assay (<50 copies/µg DNA) (**Table 2**).

**Table 2:** Detection and quantification of BLV provirus in different organs and muscle tissues of infected animals.

Sample	Arg		WT		Vac	
	nPCR	qPCR^a^	nPCR	qPCR^a^	nPCR	qPCR^a^
Supramammary lymph node	+	121,216 ± 1,053	+	53,014 ± 2,265	–	–
Prescapular lymph node	+	67,651 ± 4,706	+	48,469 ± 772	-	-
Precrural lymph node	+	70,292 ± 2,914	+	46,190 ± 545	–	<50
Spleen	+	250,552 ± 4,155	+	45,191 ± 2,625	-	<50
Liver	+	33,983 ± 3,165	+	15,303 ± 725	–	–
Uterine horn	+	17,957 ± 4	+	1,539 ± 62	-	-
Uterine body	+	13,022 ± 480	+	6,563 ± 265	–	–
Mammary gland	+	8,908 ± 837	+	720 ± 101	-	-
Bone marrow	+	8,247 ± 1,873	+	5,161 ± 1200	–	–
Heart (atrium)	+	7,386 ± 818	+	4,019 ± 429	-	-
Kidney	+	2,043 ± 700	+	1,493 ± 2	–	–
Abomasum (fundic area)	+	4,862 ± 82	+	501 ± 157	-	-
Abomasum (pyloric area)	+	2,682 ± 149	+	972 ± 26	–	–
Brain	+	2,346 ± 60	+	907 ± 13	-	-
Spinal cord	+	645 ± 31	+	715 ± 19	–	–
Cerebellum	+	653 ± 29	+	684 ± 110	-	-
Beef cuts (muscle tissue)						
Round steak	+	3,137 ± 146	+	1,418 ± 222	-	-
Blade steak	+	6,397 ± 946	+	710 ± 275	–	–
Tenderloin	+	6,317 ± 25	+	2,688 ± 279	-	-
Standing rump	+	3,503 ± 802	+	878 ± 81	–	–
Shin of beef	+	3,110 ± 160	+	1,122 ± 339	-	-
Thin skirt	+	3,100 ± 160	+	1,844 ± 918	–	–
Skirt steak	+	2,398 ± 52	+	1,306 ± 652	-	-
Flank steak	+	1,890 ± 670	+	9,353 ± 868	–	–

(+): positive (–),: not detected.

^a^ Values represent the mean ± standard deviation of proviral load (PVL) from qPCR duplicates, expressed as copies/µg DNA.

## 4 Discussion

Our study demonstrates the distribution of BLV proviral DNA across multiple organs and tissues in animals during the chronic phase of infection. Importantly, this work provides a unique long-term (seven-year) evaluation of the safety of an attenuated veterinary vaccine, addressing a critical gap in the literature where comparable safety studies are currently lacking.

Animals infected with wild-type strains (Arg and WT) exhibited high peripheral blood PVL, and proviral DNA was detected in all analyzed organs and muscle tissues. In contrast, the animal inoculated with the attenuated pBLV6073DX strain (Vac) showed very low PVL in peripheral blood, with proviral DNA detectable only by qPCR in the spleen and precrural lymph node at extremely low levels. Despite this low PVL, Vac maintained anti-BLV antibody titers which appeared sufficient to confer protection. Notably, this animal remained continuously exposed to cattle infected with wild-type BLV, yet no superinfection was detected, as nPCR consistently amplified only the vaccine strain and not the wild-type virus. During the study, antibody levels in Vac remained relatively constant, exhibiting a minor variation of approximately 2 logs, though consistently below the titers observed in Arg and WT. These findings confirm that the attenuated vaccine strain replicates at minimal levels, resulting in undetectable or very low proviral loads, while still eliciting a persistent antibody response, albeit lower than that induced by active viral replication (24).

The distribution of BLV proviral DNA in Arg and WT was consistent with previous reports from early stages of experimental infections. Klintevall et al. detected BLV proviral DNA by nPCR in several organs of calves slaughtered after 56 days of infection, most consistently in the spleen, uterus, liver, kidney, abomasum, lymph nodes and heart (35). Similarly, a recent study reported BLV proviral DNA in multiple organs of experimentally infected cows seven to nine months post. infection, with the spleen and lymph nodes showing the highest prevalence (36). Our results extend these observations, suggesting that BLV-infected cells are mostly present in these organs during long-term infection.

When PVL was compared across organs and muscle tissues, animals with high blood PVL (Arg and WT) also showed the highest PVL in the spleen and lymph nodes. Arg, which had the highest blood PVL, exhibited elevated PVL in most of the organs and muscle tissues, whereas WT, with lower blood PVL, showed correspondingly lower PVL levels. This pattern aligns with findings by Kahora et al., who demonstrated a correlation between blood PVL and the biodistribution of BLV proviral DNA in cattle during early infection (36). In agreement, the animal infected with the attenuated strain in our study presented very low blood PVL, with proviral DNA detected only in the spleen and precrural lymph node.

The predominance of PVL in the spleen and lymph nodes is expected, given their high lymphocyte content. Additionally, as the spleen retains the highest concentration of residual blood after slaughter (37) and whole-body perfusion was not performed in these adult cattle, a fraction of the detected PVL likely derives from circulating PBMCs. In the vaccinated animal, only the attenuated vaccine strain was consistently detected in peripheral blood; therefore, the proviral DNA observed in the spleen and precrural lymph node most likely reflect the presence of this same strain, although this could not be confirmed by nPCR due to the low PVL in these tissues.

In animals infected with wild-type strains, elevated PVL was also observed in the liver and uterus, organs frequently associated with BLV-induced tumors (15, 38). However, high tissue PVL during persistent infection does not automatically dictate the site of lymphosarcoma formation; for instance, while the heart is a frequent target for BLV lymphomas, cardiac PVL remained low in this study. This suggest that while physiological lymphocyte homing and residual blood account for viral detection in lymphoid tissues, tissue-specific oncogenic factors—rather than local proviral concentration alone—drive subsequent neoplastic transformation.

It has been reported that PVL levels increase with disease progression, and higher PVL is generally detected in cattle with EBL compared with BLV-infected cattle without clinical signs, both in blood (39) and in spleen and lymph nodes (40). Somura and colleagues observed that more than 70% of lymph node samples and 65% of spleen samples from EBL cattle contained over 100,000 copies/µg DNA, whereas this threshold was never exceeded in clinically healthy BLV-infected cattle (40). In our study, only the buffy coat, spleen and supramammary lymph node from Arg showed PVL values above 100,000 copies/µg DNA; however, no lesions compatible with EBL were observed at necropsy.

Although the vaccine strain is a genetically modified organism, it is important to emphasize that the attenuated strain was considered safer for the agroecosystem than the natural BLV from which it derives. This conclusion was reached by the Comisión Nacional Asesora de Biotecnología Agropecuaria (CONABIA) and the Coordinación de Innovación y Biotecnología of the Argentine Ministry of Agriculture, based on evidence generated during the vaccine efficacy and safety trial conducted at a dairy farm in Argentina (24). Moreover, although the present study involved a limited number of animals, it served as an essential proof of concept, providing critical data on the safety of the vaccine strain and supporting its deregulation process.

Over the past decade, several studies have suggested a possible association between BLV infection and human breast cancer (41–44) raising public health concerns. However, other investigations have failed to detect BLV proviral DNA in breast cancer tissue samples (45–47). According to the World Organization for Animal Health, BLV is not considered a hazard to humans (48). Although the link between BLV and breast cancer remains controversial, the use of the pBLV6073DX attenuated vaccine strain would reduce BLV prevalence and minimize any hypothetical risk of transmission to humans. The results of this study, coupled with our previous data demonstrating only low levels of vaccine proviral DNA in milk (24), significantly reinforces the safety profile of this attenuated strain regarding food products. Less controversial is the impact of BLV on economic cost and animal welfare. Our study shows that proviral loads in a cow inoculated with the BLV vaccine became extremely low in peripheral blood and undetectable in most organs. The use of this vaccine strain has the potential to reduce BLV prevalence worldwide, particularly in endemic regions where the implementation of costly control measures-such as segregation or culling of infected animals-is not feasible.

## Data Availability

The original contributions presented in the study are included in the article/**Supplementary Material**. Further inquiries can be directed to the corresponding author.
