# Supplementary figures and images for: Tissue distribution and quantification of bovine leukemia virus proviral DNA in cows after a long-term infection with wild-type strains and the attenuated BLV vaccine strain
Source: Front Immunol. 2026 Jul 15;17:1850743. doi: 10.3389/fimmu.2026.1850743 (PMC13414822; doi:10.3389/fimmu.2026.1850743)

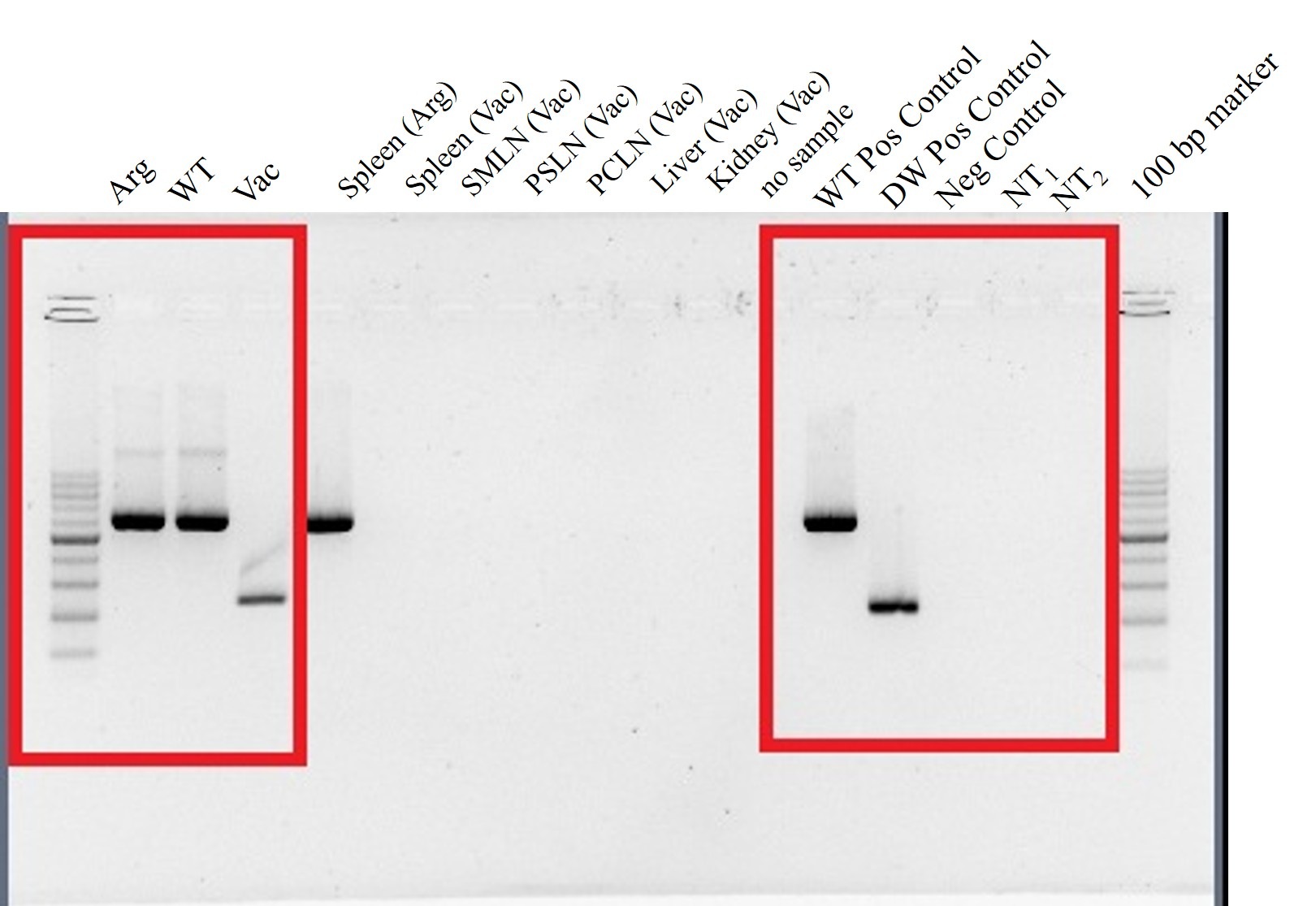

Supplement: Supplementary file 1 [file Image1.jpg]
